# Integrating single-cell and bulk RNA sequencing to predict prognosis and immunotherapy response in prostate cancer

**DOI:** 10.1038/s41598-023-42858-9

**Published:** 2023-09-20

**Authors:** Xiao Yan Wen, Ru Yi Wang, Bei Yu, Yue Yang, Jin Yang, Han Chao Zhang

**Affiliations:** 1 Department of Urology, The Affilated Hospital and Clinical Medical College of Chengdu University, No.82, North Second Section of Second Ring Road, Chengdu, 610081 Sichuan China; 2 Medical College of Soochow University, Suzhou, 215000 Jiangsu China

**Keywords:** Computational biology and bioinformatics, Immunology, Nephrology

## Abstract

Prostate cancer (PCa) stands as a prominent contributor to morbidity and mortality among males on a global scale. Cancer-associated fibroblasts (CAFs) are considered to be closely connected to tumour growth, invasion, and metastasis. We explored the role and characteristics of CAFs in PCa through bioinformatics analysis and built a CAFs-based risk model to predict prognostic treatment and treatment response in PCa patients. First, we downloaded the scRNA-seq data for PCa from the GEO. We extracted bulk RNA-seq data for PCa from the TCGA and GEO and adopted “ComBat” to remove batch effects. Then, we created a Seurat object for the scRNA-seq data using the package “Seurat” in R and identified CAF clusters based on the CAF-related genes (CAFRGs). Based on CAFRGs, a prognostic model was constructed by univariate Cox, LASSO, and multivariate Cox analyses. And the model was validated internally and externally by Kaplan–Meier analysis, respectively. We further performed GO and KEGG analyses of DEGs between risk groups. Besides, we investigated differences in somatic mutations between different risk groups. We explored differences in the immune microenvironment landscape and ICG expression levels in the different groups. Finally, we predicted the response to immunotherapy and the sensitivity of antitumour drugs between the different groups. We screened 4 CAF clusters and identified 463 CAFRGs in PCa scRNA-seq. We constructed a model containing 10 prognostic CAFRGs by univariate Cox, LASSO, and multivariate Cox analysis. Somatic mutation analysis revealed that TTN and TP53 were significantly more mutated in the high-risk group. Finally, we screened 31 chemotherapeutic drugs and targeted therapeutic drugs for PCa. In conclusion, we identified four clusters based on CAFs and constructed a new CAFs-based prognostic signature that could predict PCa patient prognosis and response to immunotherapy and might suggest meaningful clinical options for the treatment of PCa.

## Introduction

Prostate cancer (PCa) is primarily an epithelial malignancy that occurs in the prostate gland and is one of the most common cancers among men worldwide. Although the cause of PCa is not known, studies have shown that factors such as age, family history, ethnicity, and gene regulation are all involved in its development^1^. PCa exhibits geographical variability in its incidence, being more prevalent in developed nations, especially in North America and Europe, when compared to their developing counterparts. Age is a significant risk factor, with the incidence increasing substantially after the age of 50^2^. Several racial and ethnic disparities have been observed in PCa epidemiology. It is more common in African-American men compared to Caucasian men, and African-American men also tend to have a higher mortality rate associated with PCa^3^. As a consequence of the indiscernible clinical manifestations of early-stage prostate cancer, an approximate majority of patients are diagnosed belatedly, at an intermediate or advanced stage, or when distant metastases have already occurred, resulting in elevated rates of patient mortality^4^. Therefore, early screening and diagnosis of PCa are essential to reducing its mortality rate. With the rapid development of genetic analysis techniques, we have achieved a deeper understanding of the molecular pathogenesis of PCa, which in turn can be used for early diagnosis and treatment in clinics^5,6^.

The tumour microenvironment (TME) is a dynamic environment made up of multiple cells and is considered one of the most important factors influencing tumourigenesis, invasion, and metastasis^7^. Cancer-associated fibroblasts (CAFs), a major component of the TME, contribute to cancer progression by interacting with other cells to influence tumour angiogenesis and immunity evasion^8^. CAFs could induce abnormal proliferation and invasion of tumour cells by secreting large amounts of growth factors, cytokines, and chemokines and degrading extracellular matrix proteins^9^. It has been shown that CAFs are highly heterogeneous in esophageal squamous cell carcinoma and can be used as a valid predictive target for esophageal squamous cell carcinoma prognosis^10^. Studies have reported that aberrant molecular crosstalk between cancer cells and CAF is extremely important for tumour progression and metastasis, leading to poor prognosis in cancers^11,12^. In addition, a foreign study found that CAFs promoted the aggressiveness of PCa cells through mitochondrial transfer^13^. Collectively, CAFs are closely related to the development of tumours.

Despite the voluminous body of research on CAFs carried out by a plethora of scholars, a comparatively insufficient comprehension of the properties of CAFs persists, including their correlation with prostate cancer prognosis and immunotherapy. With advances in sequencing technology, increasing numbers of scholars are turning their attention to single-cell RNA-sequencing (scRNA-seq). The scRNA-seq technology could focus on detecting the expression of single cell subpopulations in tissues, bridge the shortage of traditional sequencing technologies, and help us further explore and construct the gene expression profiles of various cells to better understand the pathological processes of diseases^14^. The scRNA-seq can analyze the composition and gene expression of thousands of individual cells at high resolution, making it better suited for exploring tumour heterogeneity^15^. More and more researches are combining RNA-seq and scRNA-seq data to analyze tumor heterogeneity and construct models and nomograms to predict cancer patient prognostic outcomes, thus providing new approaches to tumor clinical treatment^16,17^. A study demonstrated that significant heterogeneity within CAFs was found in human PCa tissue and revealed that CAF subpopulations stimulate the growth and development of PCa by aberrant recruitment of immune cells^18^. However, at present, there are relatively few scRNA-seq analyses focused on exploring the correlation between CAFs and PCa. Therefore, we have explored the relationship between PCa and CAFs by combining bulk RNA-seq and scRNA-seq techniques to provide new ideas for the clinical diagnosis and treatment of PCa.

In the study, we acquired the scRNA-seq and RNA-seq data for PCa from the GEO and TCGA datasets and conducted batch correction to merge the two datasets. Then we analyzed the scRNA-seq data, constructed a prognostic model associated with PCa, and validated the model. We further identified differentially expressed genes (DEGs) between risk groups and performed Gene Ontology (GO) and Kyoto Encyclopedia of Genes and Genomes (KEGG) analyses. We also explored differences in somatic mutations between different risk groups. Finally, we filtered 31 chemotherapeutic agents and targeted therapeutics that are closely related to PCa. Overall, our study may provide novel thinking on the pathogenesis of PCa and new perspectives for the personalized treatment of PCa patients.

## Methods

### Data download and organization

The scRNA-seq data for PCa were acquired from the GEO-GSE141445, including 36,424 cells from 13 PCa samples^19^. The RNA-seq data and clinical information for PCa were acquired from the TCGA-PRAD, GEO-GSE46602, GEO-GSE70769, and GEO-GSE116918^20–22^. After removing data with missing or less than 30 days of follow-up, missing outcome time occurrence status, and duplicates, the TCGA-PRAD set remained at 494 PCa samples, the GEO-GSE46602 set remained at 36 PCa samples, the GEO-GSE70769 set remained at 92 PCa samples, and the GSE116918 set remained at 248 PCa samples. As only the TCGA-PRAD and GSE116918 datasets retained the complete clinical data, we merged the two datasets into a whole set and used the function "ComBat" to remove the batch effect^23,24^.

### Analytical process for scRNA-seq data

Firstly, we created a Seurat object for the scRNA-seq data using the R package “Seurat” (4.3.0)^25^. The quality control criteria we added according to the previous threshold only excluded cells with a hemoglobin ratio greater than 1^19^. Then, we performed cell cycle effects removal, normalization, dimensionality reduction (1:30), clustering (resolution = 0.5) and cell annotation on the Seurat objects^19,26^. To overcome the extensive technical noise in any single feature for scRNA-seq data, Seurat clusters cells based on their PCA scores, with each PC essentially representing a ‘metafeature’ that combines information across a correlated feature set. Generally we selected the cumulative variance contribution to be just over 85% of the total variance, so we selected the top 30 PCs. Finally, we extracted the fibroblasts and repeated the previous analysis, using the function "FindAllMarkers" to find the high variant genes in each cluster (logFC > = 0.5, min.pct = 0.3, and diff.pct > = 0.2), and using the R package "clusterProfiler" to perform enrichment analysis of the high variant genes in each cluster (*p* < 0.05)^27^. These highly variable genes were identified as CAF-related genes (CAFRGs).

### Construction of prognostic model

We randomly split the samples from the whole set into a train set and a test set according to a 7:3 ratio using the R package "caret". To identify CAFRGs that have significant prognostic value, we conducted a univariate Cox analysis on these genes. This statistical analysis allowed us to explore the correlation between the expression of CAFRGs and patient outcomes, which in turn enabled us to identify specific genes that were associated with a higher risk of disease progression or mortality. In order to refine our selection of prognostically significant CAFRGs, we conducted a LASSO analysis. This approach involves a regularization technique that seeks to reduce the number of variables used in a model while maintaining its predictive power by shrinking the coefficients of less relevant genes towards zero. In addition, we used tenfold cross-validation to assess the performance of our LASSO model and ensure that it was not overfitting our dataset. By employing this method, we were able to further compress the list of CAFRGs to a smaller set of genes that exhibited the strongest prognostic value in our study^28^. To develop a robust prognostic model for cancer patients, we employed a stepwise multivariate Cox analysis. This analysis allowed us to examine the impact of multiple prognostic factors on survival outcomes, taking into account the potential interactions between these factors. The scores for each sample were computed in accordance with the subsequent equation: risk score = coefficient1 * gene1 expression + … + coefficientN * geneN expression, and the samples were stratified into high-risk and low-risk groups using the median score as the cutoff point..

### Validation of prognostic signature

Kaplan–Meier analysis with a chi-squared test was applied to estimate survival differences between risk groups in the model. The test set, whole set, TCGA-PRAD set, and GSE116918 set were applied to validate the internal stability of the model. Although the GSE46602 set and GSE70769 set lacked survival status and time, the GSE46602 set, GSE70769 set, and GSE116918 set contained the status and time of biochemical recurrence (BCR). Therefore, we exploited the model to predict the BCR probability of the GSE46602 set, GSE70769 set, and GSE116918 set. The time-dependent receiver operating characteristic (ROC) curves were employed to analyze the predictive performance of the model and clinical characteristics through the R package “timeROC”^29^. To assess whether the model was an independent predictor for predicting patient prognosis, we performed univariate and multivariate Cox analyses, including risk scores and clinical characteristics. To better apply the model to clinical work, we constructed a nomogram combining the model and clinical features to predict the 3-, 5-, and 7-year survival probabilities of patients.

### Enrichment analysis and somatic mutations

To find differences in molecular mechanisms and relevant pathways between risk groups, we recognized DEGs between risk groups (|logFC > 1| and FDR < 0.05) and conducted GO and KEGG analyses (*p* < 0.05)^24,27,30,31^. To investigate the dissimilarities of somatic mutations among divergent risk strata, we carried out comprehensive computational and graphical exploration utilizing the R software library "maftools"^32^.

### Immune microenvironment and immunotherapy

To explore the landscape of the immune microenvironment in the different groups, we used the single-sample Gene Set Enrichment Analysis (ssGSEA) algorithm to score the immune cells and immune function in each sample and tested both groups using the Wilcoxon test^33,34^. We also explored the differences in immune checkpoint gene (ICG) expression levels between groups using the Wilcox test. To predict the response to immunotherapy in each risk group, we calculated the tumor mutation burden (TMB) scores for each group and compared them^35^.

### Development of individualized anti-tumor treatment protocols

In order to customize treatment regimens for patients belonging to distinct risk categories, we computed the half inhibitory concentration (IC50) values of the drugs employing the R software package "pRRophetic"^36^. We further analyzed and compared the differences in the IC50 values between the different risk cohorts via the Wilcoxon rank-sum test, yielding a statistically significant p-value of < 0.001.

## Result

### Identification of CAF

**Figure 1:**
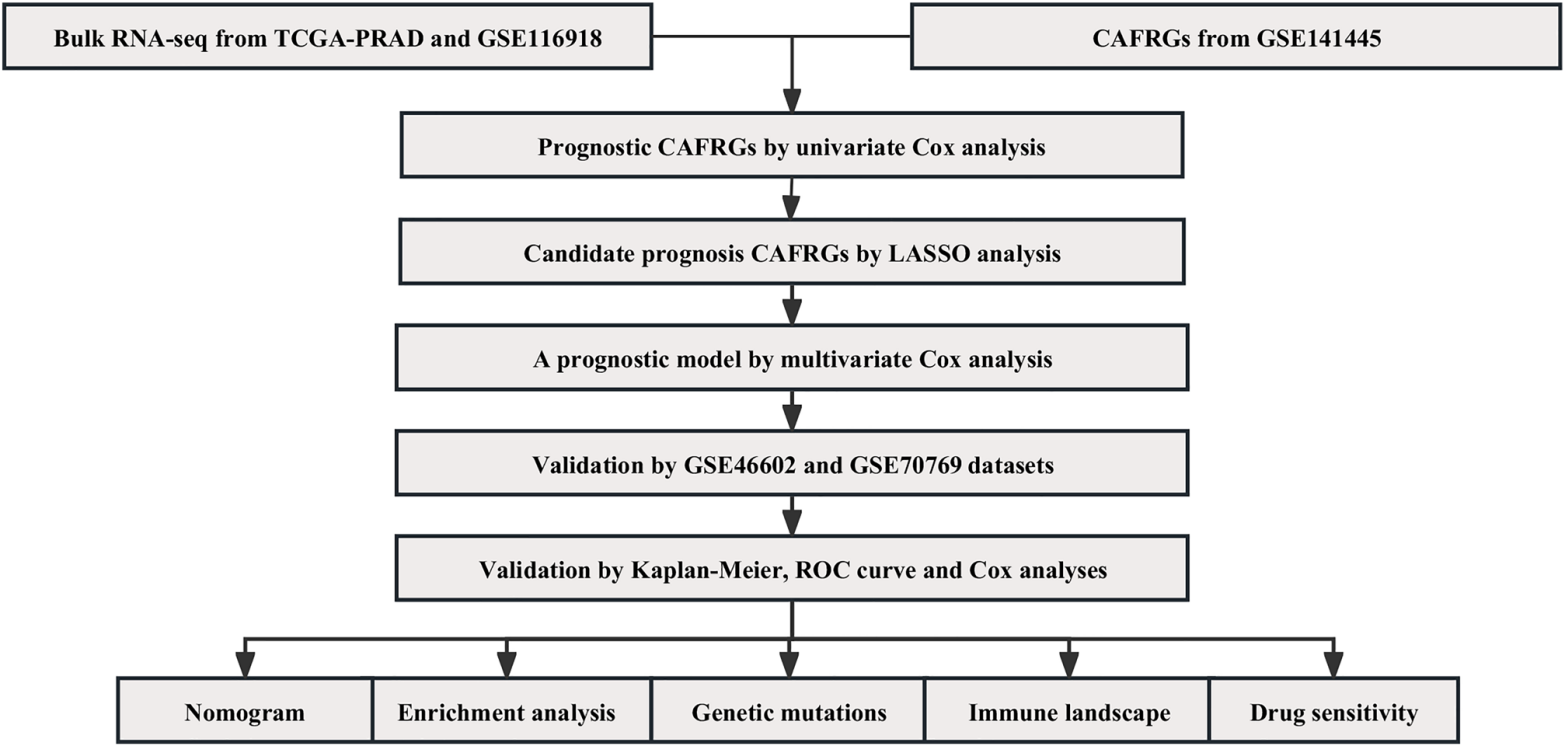
The overall workflow.

**Figure 2:**
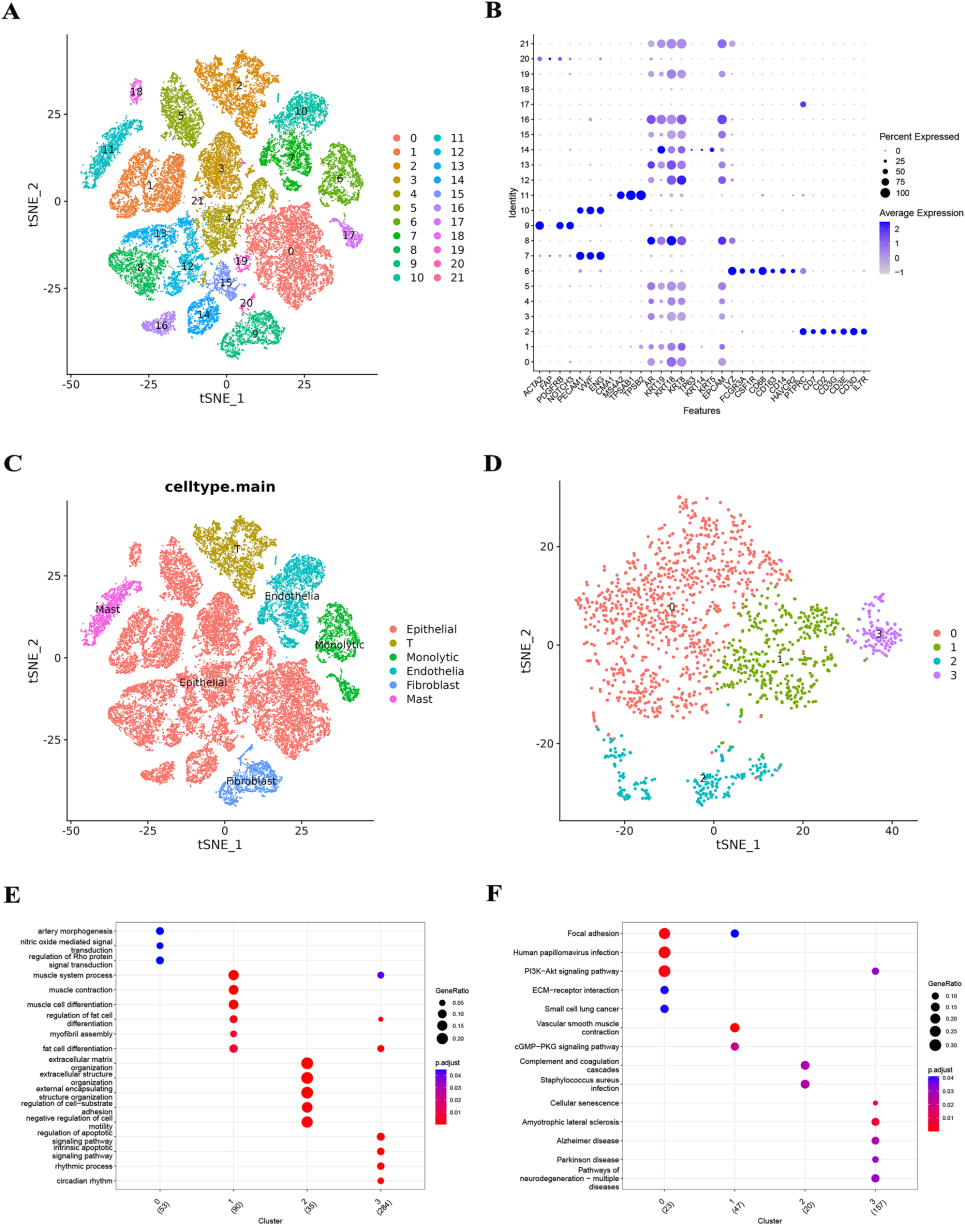
(**A**) The tSNE plot showed the distribution of different clusters. (**B**) The bubble plots showed the expression levels of marker genes in each cluster. (**C**) Cell annotations for these clusters. (**D**) The tSNE plot showed the distribution of the four clusters for CAF. (**E** and **F**) The GO and KEGG analyses for 463 CAFRGs in different CAF clusters.

The workflow of this study is summarized in Fig. 1. We performed cell cycle effect removal, normalization, dimensionality reduction, and clustering on the scRNA-seq data, and the tSNE plot showed the distribution of different clusters (Fig. 2A). The bubble plots showed the expression levels of marker genes in each cluster (Fig. 2B) and we performed cell annotations for these clusters (Fig. 2C). We extracted fibroblasts and repeated the above analysis on them, and the tSNE plot showed the distribution of the four clusters (Fig. 2D). We identified a total of 463 CAFRGs using the function "FindAllMarkers" for the high variant genes in each cluster (logFC >= 0.5, min.pct = 0.3 and diff.pct >= 0.2) (Supplementary Table S1). Finally, the enrichment analysis showed that cluster 0 artery morphogenesis, nitric oxide mediated signal transduction, regulation of Rho protein signal transduction, PI3K-Akt signaling pathway, and ECM-receptor interaction, cluster 1 is mainly enriched in muscle system process, muscle contraction, muscle cell differentiation, vascular smooth muscle contraction, and cGMP-PKG signaling pathway, cluster 2 is mainly enriched in extracellular matrix organization, extracellular structure organization, external encapsulating structure organization, complement and coagulation cascades, and staphylococcus aureus infection, and cluster 3 is mainly enriched in regulation of apoptotic signaling pathway, intrinsic apoptotic signaling pathway, rhythmic process, cellular senescence, and amyotrophic lateral sclerosis **(**Fig. 2E and F), as detailed in Supplementary Tables S2 and S3.

### Development of prognostic model

**Figure 3:**
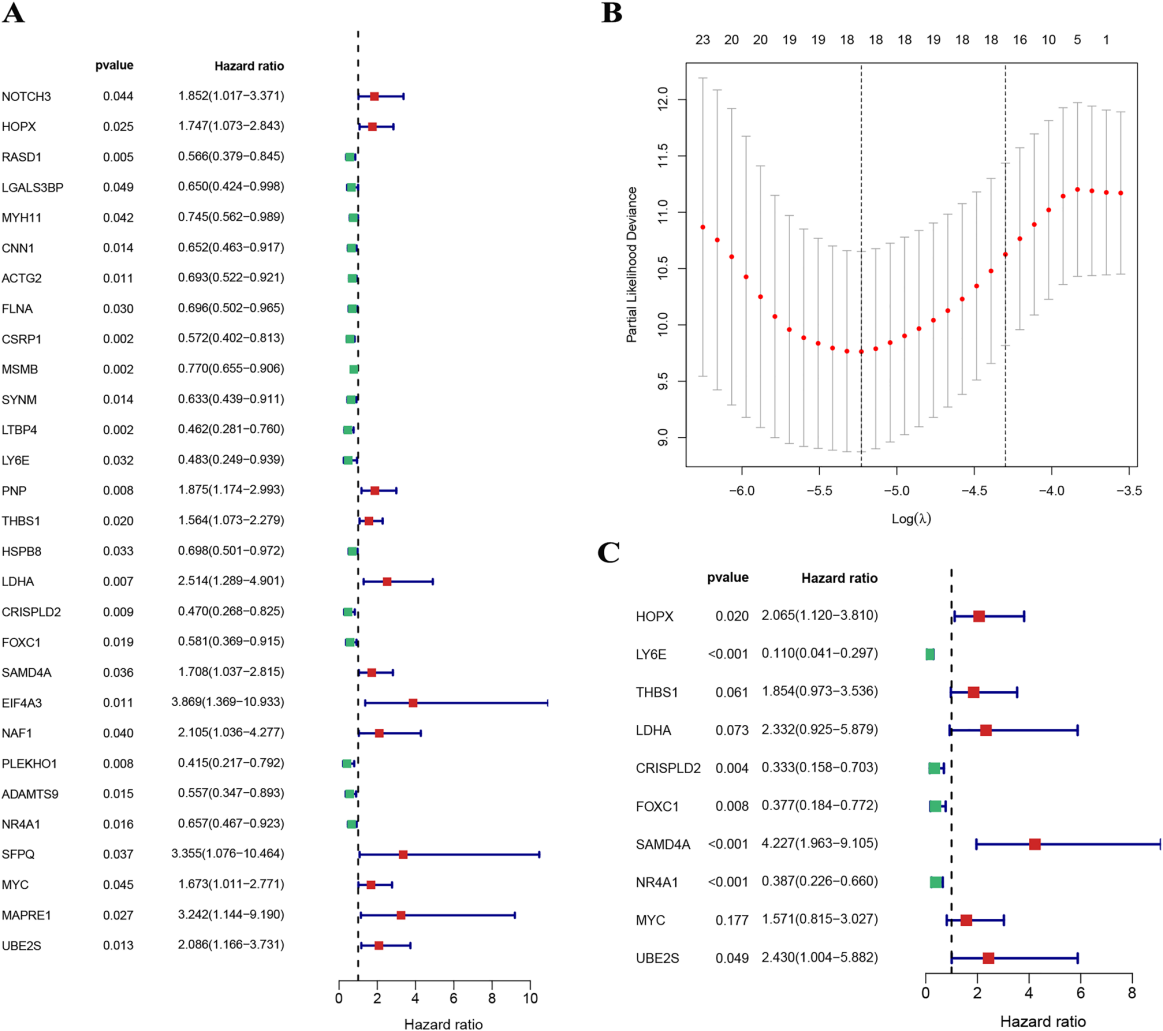
(**A**) The univariate Cox analysis screened 29 prognostic CAFRGs. (**B**) The LASSO analysis further screened 18 candidate CAFRGs. (**C**) The multivariate Cox analysis constructed a model containing 10 prognostic CAFRGs.

We randomly split the PCa samples from the whole set into a train set and a test set according to a 7:3 ratio; the train set contained 522 PCa samples, and the test set contained 220 PCa samples. The univariate Cox analysis screened 29 prognostic CAFRGs (Fig. 3A), the LASSO analysis further screened 18 candidate CAFRGs (Fig. 3B), and the multivariate Cox analysis constructed a model containing 10 prognostic CAFRGs (Fig. 3C).

### Validation of prognostic signature

**Figure 4:**
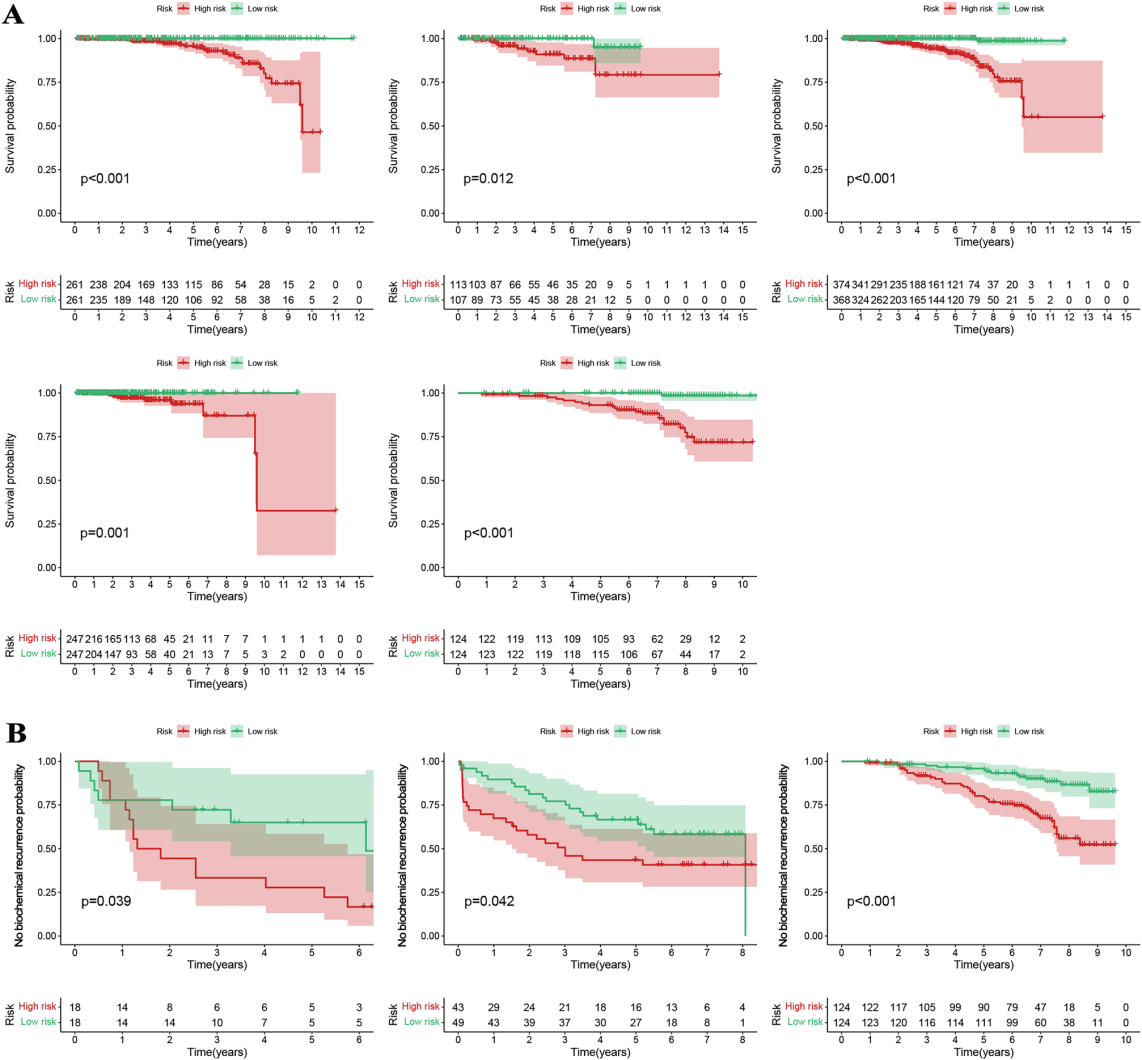
(**A**) The survival rates of PCa patients in the low-risk group were significantly higher than those in the high-risk group for different internal sets (the train, test, overall, TCGA, and GSE116918 sets). (**B**) PCa patients in the high-risk group are more likely to had a higher BCR under different external groups (the GSE46602, GSE70769, and GSE116918 sets).

**Figure 5:**
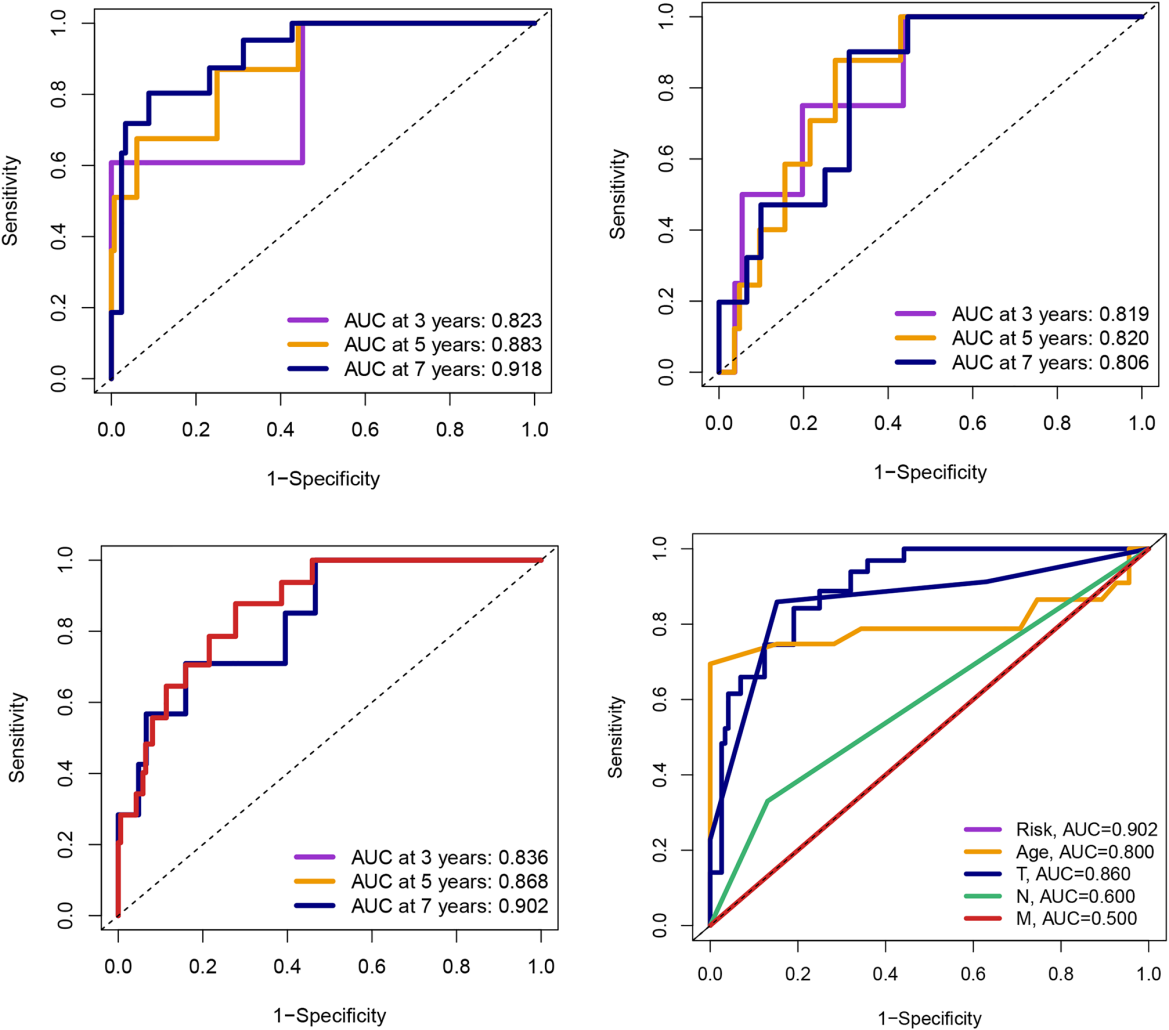
All AUC values for the signature were higher than 0.8 and greater than the AUC values for clinical features.

**Figure 6:**
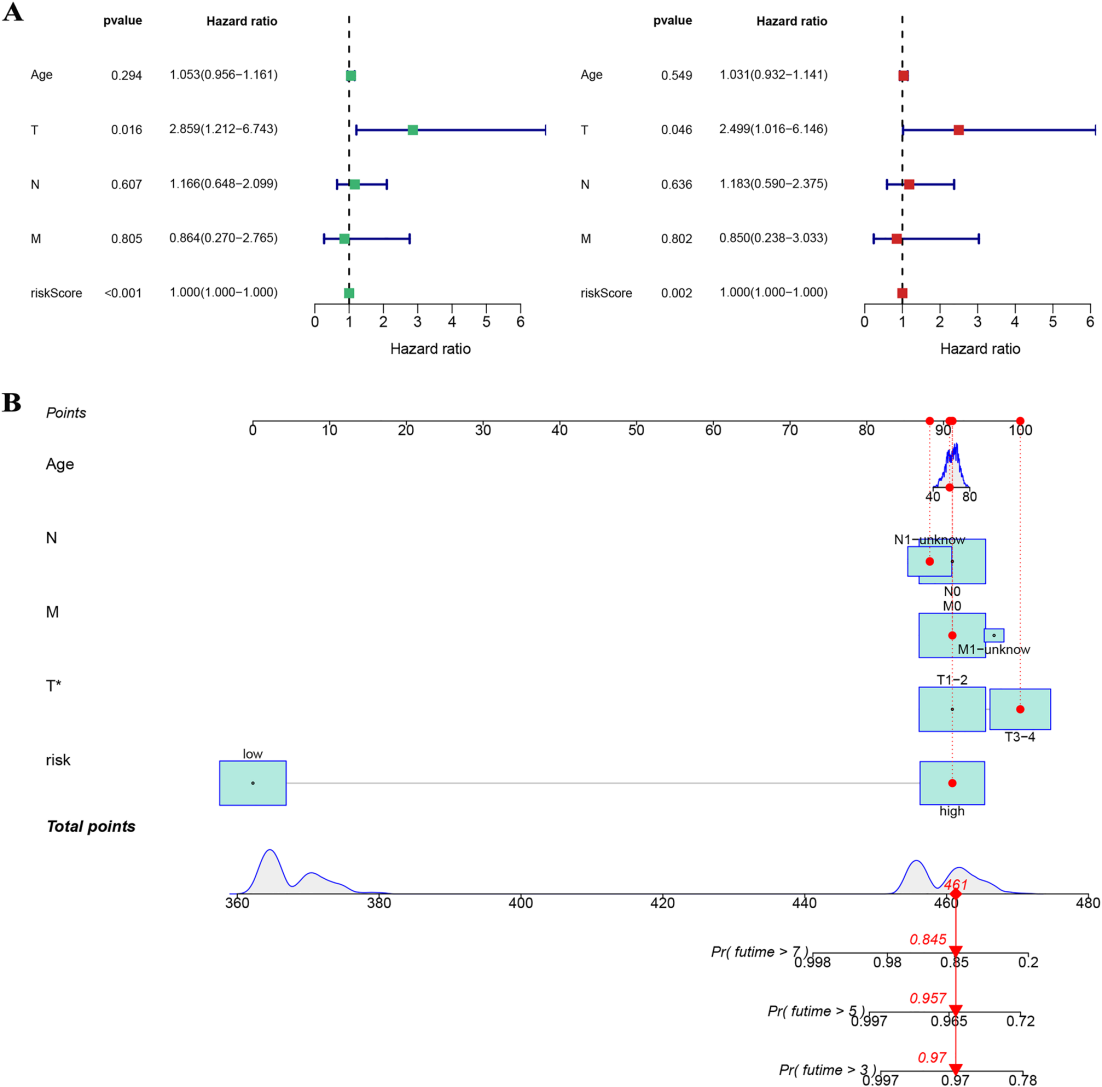
(**A**) The model was an independent predictor for predicting PCa patient prognosis by univariate and multivariate Cox analyses. (**B**) A nomogram combining the model and clinical characteristics to predict the 3-, 5-, and 7-year survival probabilities of PCa patients.

Figure 4A showed that the survival rates of PCa patients in the low-risk group were significantly higher for different internal sets (the train, test, overall, TCGA and GSE116918 sets) and the differences were statistically different, indicating that the stability of the model was high internally. Figure 4B showed that PCa patients in the high-risk group are more likely to have a higher BCR under different external groups (the GSE46602, GSE70769 and GSE116918 sets), and the differences were statistically different, indicating that the model is able to predict not only the prognosis but also the BCR for PCa patients. All area under curve (AUC) values for the signature were higher than 0.8 and greater than the AUC values for clinical features (Fig. 5). The model was an independent predictor for predicting PCa patient prognosis (Fig. 6A). To better apply the model in clinical work, we constructed a nomogram combining the model and clinical characteristics to predict the 3-, 5-, and 7-year survival probabilities of PCa patients, indicating that the high- or low-risk groups had the greatest impact on prognosis (Fig. 6B).

### Enrichment analysis and somatic mutations

**Figure 7:**
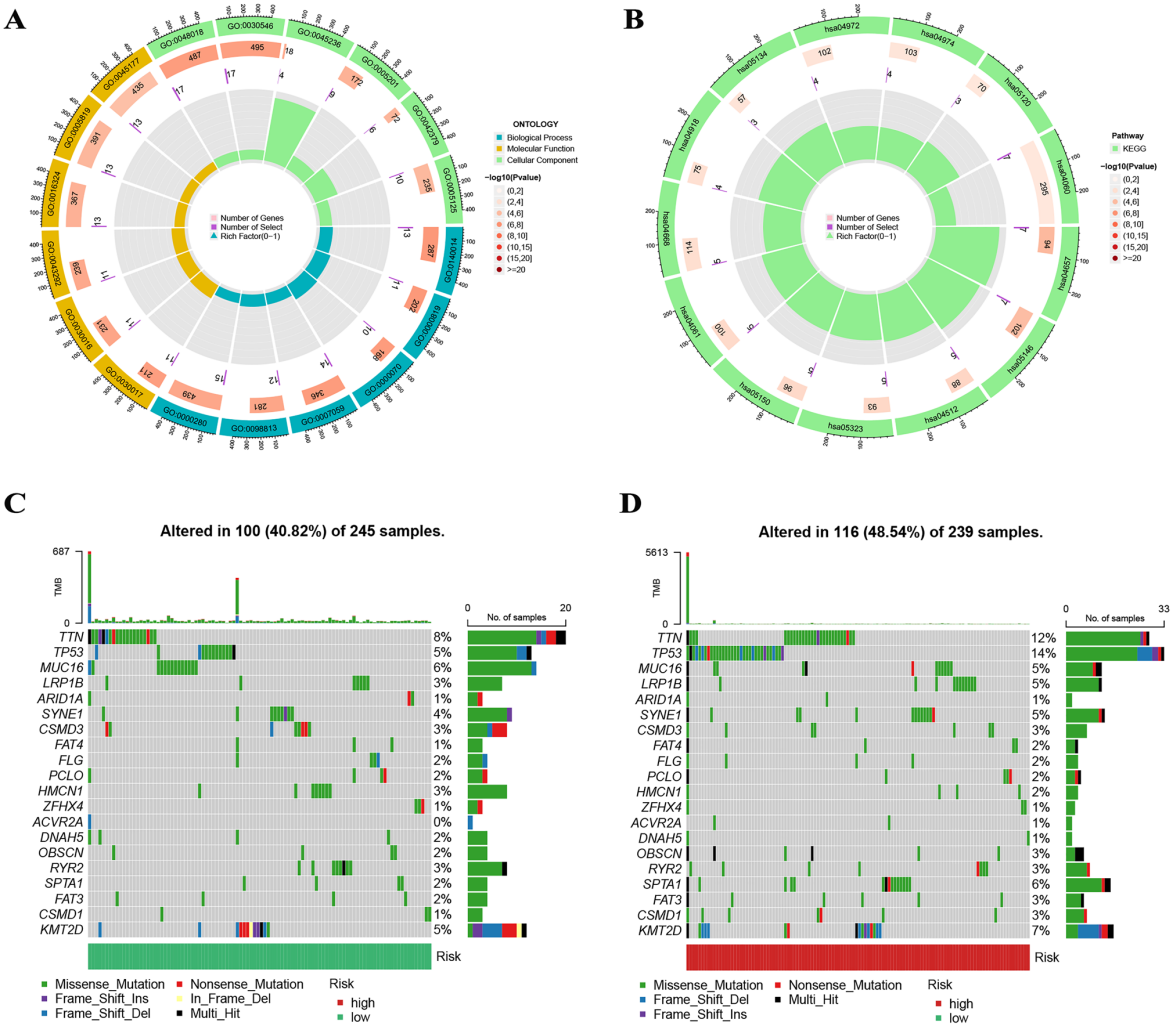
(**A** and **B**) The GO and KEGG analyses for different CAF clusters. (**C** and **D**) More mutations in the high-risk group.

183 DEGs were identified between different groups with thresholds of |logFC > 1| and FDR < 0.05 (Supplementary Table S4). GO and KEGG analyses were employed to explore the role and molecular pathways of these DEGs in PCa. Biological process (BP) terminology is associated with mitotic nuclear division (GO:0140014), sister chromatid segregation (GO:0000819), and mitotic sister chromatid segregation (GO:0000070); cellular component (CC) terminology is associated with sarcomere (GO:0030017), myofibril (GO:0030016), and contractile fiber (GO:0043292); molecular function (MF) terms is associated with receptor ligand activity (GO:0048018), signaling receptor activator activity (GO:0,030,546), CXCR chemokine receptor binding (GO:0045236) (Fig. 7A and Supplementary Table S5). KEGG analysis showed that DEGs were highly enriched in the IL-17 signaling pathway (hsa04657), amoebiasis (hsa05146), ECM-receptor interaction (hsa04512), rheumatoid arthritis (hsa05323), staphylococcus aureus infection (hsa05150), and viral protein interaction with cytokine and cytokine receptor (hsa04061) (Fig. 7B and Supplementary Table S6). Figures 7C and D showed the gene mutations in different risk groups, indicating that the high-risk group has significantly more mutations than the low-risk group, which may be related to the poor prognosis in the high-risk group.

### Immune microenvironment and immunotherapy

**Figure 8:**
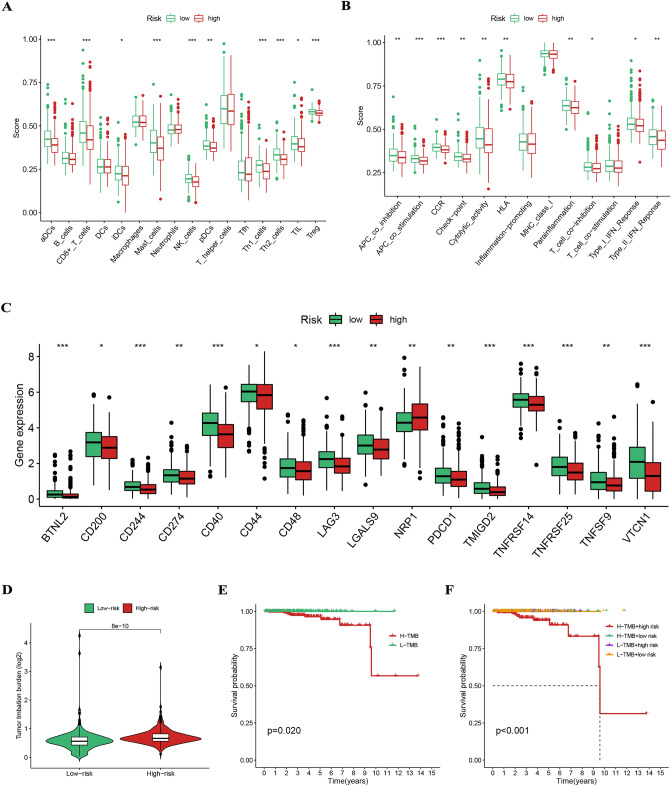
(**A**) Most immune cell infiltration levels were higher in the low-risk group. (**B**) Immune function scores were higher in the low-risk group, except for inflammation promoting, major histocompatibility complex class I and T cell co-stimulation. (**C**) The expression of ICGs such as CD274, LAG3, and PDCD1 differed between the different risk groups. (**D**) The higher TMB scores in the high-risk group. (E and F) The TMB score combined with the risk score was also a strong predictor of patient prognosis.

Figure 8A indicated that most immune cell infiltration levels were higher in the low-risk group. Figure 8B indicated that immune function scores were higher in the low-risk group, except for inflammation promoting, major histocompatibility complex class I and T cell co-stimulation. The expression of ICGs such as CD274, LAG3, and PDCD1 also differed between the different risk groups (Fig. 8C). Theoretically, the higher the TMB score, the more neoantigens can be recognized by T cells and, therefore, the better the immunotherapy effect. The higher TMB scores in the high-risk group indicated that patients in the high-risk group are more suitable for immunotherapy (Fig. 8D). And the TMB score combined with the risk score was also a strong predictor of patient prognosis (Fig. 8E and F).

### Finding anti-tumor drugs for patients in different risk groups

**Figure 9:**
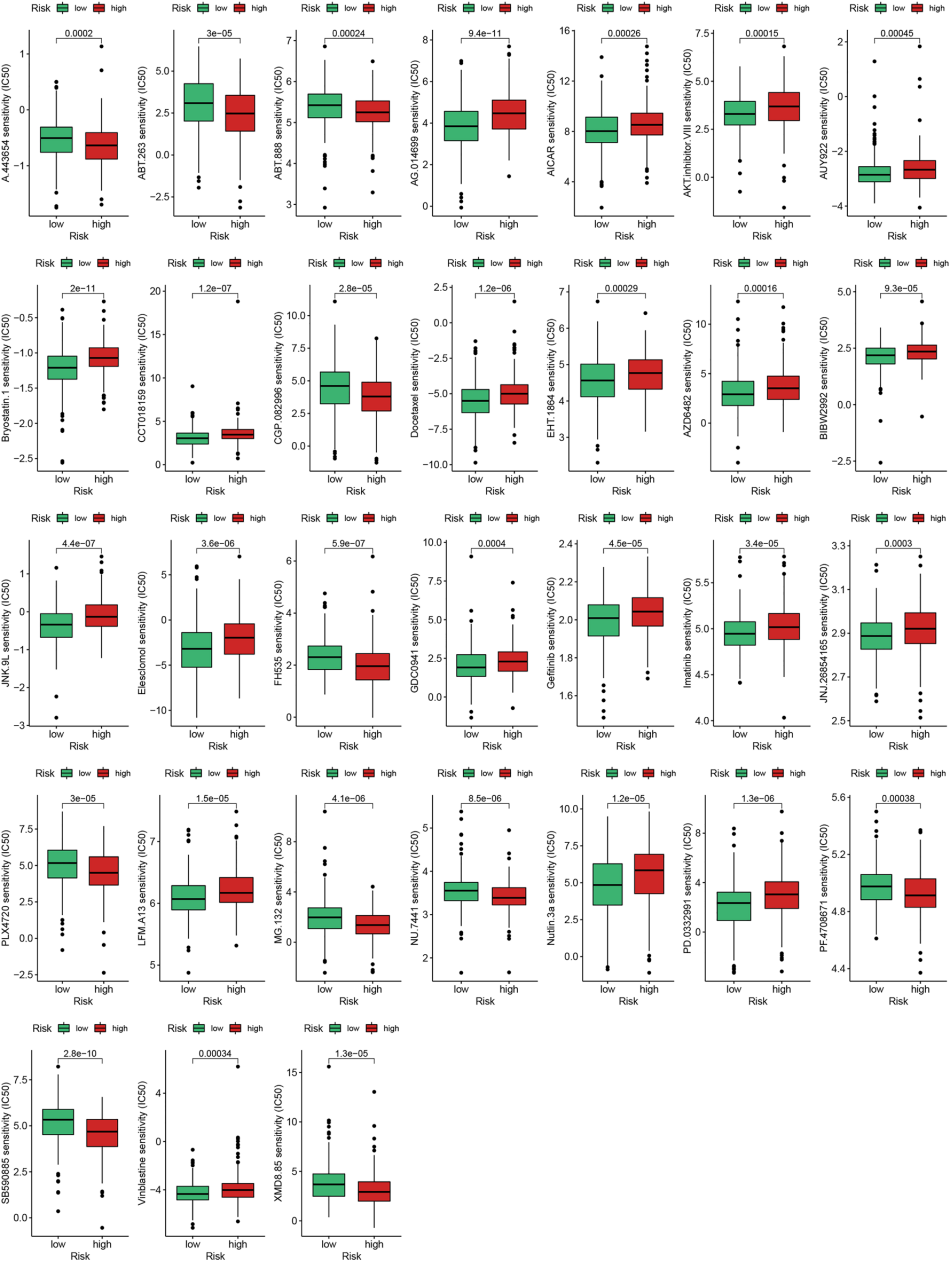
The IC50 of different groups by the R package "pRRophetic", and 31 chemotherapeutic drugs and targeted therapeutic drugs.

In addition to immunotherapy, chemotherapy and targeted therapy are also the main strategies for the treatment of PCa. In this study, we calculated the IC50 of different groups using the R package "pRRophetic", and finally screened 31 chemotherapeutic drugs and targeted therapeutic drugs (*p* < 0.001; Fig. 9).

## Discussion

PCa is one of the leading diseases affecting men's health around the world, and its incidence has been increasing in recent years^37^. The current treatment of PCa is mainly based on hormonal therapy, radiotherapy, and chemotherapy, but the majority of patients have a worse prognosis and mortality remains high^38^. As a consequence, there is a pressing demand in clinical practice to seek novel therapeutic strategies to develop individualized treatments for PCa patients in order to improve their prognosis. Numerous studies have confirmed that dynamic communication between tumour cells and CAFs accelerates the growth and progression of tumours^39,40^. With the development of biotechnology, single-cell sequencing analysis can help us gain comprehension the mechanism of the role of CAFs in the pathological progression of PCa and more accurately search for potential new biomarkers, thus effectively improving therapeutic outcomes.

As tumour heterogeneity is quite pronounced, the response of the same stage patients to the same treatment methods has a strongly variable individual outcome and affects the prognosis of the patients^41^. Research on PCa has found significant differences in outcomes for patients with similar clinical stages who receive the same therapeutic approach^42^. Therefore, a more extensive investigation into the association between the diversity of tumour characteristics in PCa and its corresponding implications on treatment response and patient prognoses is warranted. In our study, based on scRNA-seq data, we divided the PCa cells into 4 clusters, screened 463 CAFRGs, and performed functional enrichment analysis of CAFRGs. On the basis of the screening above, we constructed a prognostic model for PCa incorporating 10 CAFRGs by using multivariate Cox analysis. Next, we validated the reliability and stability of the prognostic model using multiple datasets for internal and external validation, respectively. We investigated differences in somatic mutations between high-risk and low-risk groups. We also explored differences in the immune microenvironment landscape and ICG expression levels in the different groups. Lastly, we predicted the therapeutic response of PCa and searched for anti-tumour drugs by calculating the IC50 in different groups.

For our constructed prognostic model, we found that most of the 10 CAFRGs were involved in the pathogenesis of PCa. THBS1 could accelerate malignant cancer cell invasion in PCa and promote melanoma metastasis^43,44^. LDHA is highly expressed in PCa, and inhibition of LDHA can delay the progression of PCa^45^. A study found that MIR-138-5P could inhibit the development of PCa by targeting FOXC1^46^. The expression level of NR4A1 in PCa tissue is significantly elevated in comparison to that in the normal surrounding tissue^47^. MYC promoted PCa oncogenic signaling via the PI3K/AKT/mTOR pathway, thereby stimulating massive cancer cell growth^48^. The most recent investigation has established that UBE2S, an E2 ubiquitin-conjugating enzyme, plays a critical role in stabilizing β-linked proteins via K16-linked ubiquitination, which is known to trigger enhanced migration and invasion of tumor cells in the context of prostate cancer bone metastases and promote oncogenic activities. Furthermore, it has been suggested that UBE2S-mediated ubiquitination of β-linked proteins may contribute to tumorigenesis and the progression of PCa bone metastases by facilitating alterations in key signaling pathways that promote tumor cell survival and proliferation^49^. Although there is no direct evidence linking HOPX, LY6E, SAMD4A, and CRISPLD2 to the development of PCa, numerous studies have shown their close relationship with the growth and invasion of other tumours^50–53^. The aforementioned findings have the potential to serve as novel biomarkers that can aid in the development of new therapeutic approaches for the treatment of PCa.

TP53 is a tumour suppressor gene that normally acts as a guardian to protect the organism^54^. When TP53 is mutated, the body loses its protection, which may lead to uncontrolled tumour growth. Growing evidence has demonstrated the prevalence of mutated TP53 in cancer and its association with cancer susceptibility and drug resistance^55^. The risk of aggressive PCa may be higher in men who have mutations in the TP53 gene^56^. Our analysis results are generally consistent with previous studies. TTN is a commonly mutated gene in cancer and has a prominent role in predicting immune checkpoints and prognosis in solid tumours^57^. TTN mutations have been shown to be an independent risk factor for thyroid cancer and to predict a poorer prognosis for patients^58^. The sequencing data of lung adenocarcinoma patients showed that patients with TTN gene mutations had higher immunogenicity, immune scores, and inflammatory TME, which stimulates tumour growth^59^. Nevertheless, the current number of studies on TTN in PCa is limited. Our study elucidates some references for the role of TTN in PCa.

The TME is a complex structure that provides the environment for tumor cell proliferation, invasion, and metastasis based on the presence of a large number of immune cells and immunomodulatory molecules in the tumor microenvironment^60^. Our results revealed that the majority of immune cell infiltration levels were higher in the low-risk group. Significant increases in Treg cells have been found in many malignant cancers^61^. NK cells are core cells of the natural immune system that have been found to be abnormally expressed in prostate tumours, resulting in more rapid tumour development^62^. Breakthroughs have been made in NK cell-based cancer treatment, but there are still relatively few treatment strategies for PCa^63^. Our findings may provide new inspiration for the utilization of NK cells in the treatment of PCa. And we found that ICGs such as CD274, LAG3, and PDCD1 expression differed between high- and low-risk groups. A study revealed that the CD274 (PD-L1) gene could stimulate the immune escape of tumor cells^64^. High expression of LAG3 in peripheral blood T cells and lymphocytes is strongly correlated with a worse prognosis in PCa^65^. The above studies are broadly consistent with our findings, demonstrating the reliability of our outcomes.

Despite the notable findings reported in this study, it is important to acknowledge certain limitations that may impact the interpretation of the results. Firstly, our analysis data is drawn from public datasets, and the results may be influenced by the datasets themselves, resulting in some possible bias in the results. Thus, we adopted several datasets for multiple validations from internal and external validation datasets, respectively, to support the reliability of the prognostic model we built. Secondly, we included a relatively smaller sample size of PCa, which may lead to analyses with some bias. Thirdly, due to the difficulty of collecting PCa tissue samples, we have not conducted experimental validation for the moment. In the future, we will endeavor to experimentally validate the results of our analyses, which will be the main direction of our future work.

## Conclusion

Herein, we analyzed CAFs from PCa single-cell sequencing data, grouped them into four clusters, and identified CAFRGs. We also constructed a nomogram of PCa prognosis based on CAFRGs to predict patient prognosis and response to immunotherapy. In summary, our study will provide a novel clinical perspective on the targeted treatment of PCa and better predict the prognostic outcome of patients.

### Supplementary Information

Supplementary Legends.

Supplementary Table S1.

Supplementary Table S2.

Supplementary Table S3.

Supplementary Table S4.

Supplementary Table S5.

Supplementary Table S6.

## Data Availability

The datasets used and/or analysed during the current study are available from the corresponding author on reasonable request.

## References

[1] Wang G, Zhao D, Spring DJ, DePinho RA (2018). Genetics and biology of prostate cancer. Genes Dev..

[2] Sung H (2021). Global Cancer Statistics 2020: GLOBOCAN Estimates of Incidence and Mortality Worldwide for 36 Cancers in 185 Countries. CA Cancer J. Clin..

[3] Hassanipour S (2020). Survival rate of prostate cancer in Asian countries: A systematic review and meta-analysis. Ann. Glob. Health.

[4] Pishgar F, Ebrahimi H, Saeedi MS, Fitzmaurice C, Amini E (2018). Global, Regional and National Burden of Prostate Cancer, 1990 to 2015: Results From the Global Burden of Disease Study 2015. J. Urol..

[5] Bijnsdorp IV, van Royen ME, Verhaegh GW, Martens-Uzunova ES (2017). The non-coding transcriptome of prostate cancer: Implications for clinical practice. Mol. Diagn. Ther..

[6] He S (2019). The expression of miR-375 in prostate cancer: a study based on GEO, TCGA data and bioinformatics analysis. Pathol. Res. Pract..

[7] Agnes A, Biondi A, Laurino A, Persiani R, D'Ugo D (2020). Global updates in the treatment of gastric cancer: A systematic review. Part 1: staging, classification and surgical treatment. Updates Surg..

[8] Biffi G, Tuveson DA (2021). Diversity and biology of cancer-associated fibroblasts. Physiol. Rev..

[9] Chatterjee A (2019). MicroRNA-222 reprogrammed cancer-associated fibroblasts enhance growth and metastasis of breast cancer. Br. J. Cancer.

[10] Cheng Y (2015). Cancer-associated fibroblasts are associated with poor prognosis in esophageal squamous cell carcinoma after surgery. Int. J. Clin. Exp. Med..

[11] Sunami Y, Haussler J, Zourelidis A, Kleeff J (2022). Cancer-associated fibroblasts and tumor cells in pancreatic cancer microenvironment and metastasis: Paracrine regulators, reciprocation and exosomes. Cancers (Basel).

[12] Wong KY (2022). Cancer-associated fibroblasts in nonsmall cell lung cancer: from molecular mechanisms to clinical implications. Int. J. Cancer..

[13] Ippolito L (2019). Cancer-associated fibroblasts promote prostate cancer malignancy via metabolic rewiring and mitochondrial transfer. Oncogene.

[14] Yu X, Xu X, Zhang J, Li X (2023). Batch alignment of single-cell transcriptomics data using deep metric learning. Nat. Commun..

[15] Papalexi E, Satija R (2018). Single-cell RNA sequencing to explore immune cell heterogeneity. Nat. Rev. Immunol..

[16] Kang Z, Wang J, Huang W, Liu J, Yan W (2022). Identification of transcriptional heterogeneity and construction of a prognostic model for melanoma based on single-cell and bulk transcriptome analysis. Front. Cell Dev. Biol..

[17] Yin J (2022). Identification of molecular classification and gene signature for predicting prognosis and immunotherapy response in HNSCC using cell differentiation trajectories. Sci. Rep..

[18] Vickman RE (2020). Heterogeneity of human prostate carcinoma-associated fibroblasts implicates a role for subpopulations in myeloid cell recruitment. Prostate.

[19] Chen S (2021). Single-cell analysis reveals transcriptomic remodellings in distinct cell types that contribute to human prostate cancer progression. Nat. Cell Biol..

[20] Ross-Adams H (2015). Integration of copy number and transcriptomics provides risk stratification in prostate cancer: A discovery and validation cohort study. EBioMedicine.

[21] Mortensen MM (2015). Expression profiling of prostate cancer tissue delineates genes associated with recurrence after prostatectomy. Sci. Rep..

[22] Jain S (2018). Validation of a metastatic assay using biopsies to improve risk stratification in patients with prostate cancer treated with radical radiation therapy. Ann. Oncol..

[23] Johnson T (2007). Bayesian method for gene detection and mapping, using a case and control design and DNA pooling. Biostatistics.

[24] Ritchie ME (2015). Limma powers differential expression analyses for RNA-sequencing and microarray studies. Nucleic Acids Res..

[25] Hao Y (2021). Integrated analysis of multimodal single-cell data. Cell.

[26] Hafemeister C, Satija R (2019). Normalization and variance stabilization of single-cell RNA-seq data using regularized negative binomial regression. Genome Biol..

[27] Wu T (2021). ClusterProfiler 4.0: A universal enrichment tool for interpreting omics data. Innovation (Camb.).

[28] Friedman J, Hastie T, Tibshirani R (2010). regularization paths for generalized linear models via coordinate descent. J. Stat. Softw..

[29] Blanche P, Dartigues JF, Jacqmin-Gadda H (2013). Estimating and comparing time-dependent areas under receiver operating characteristic curves for censored event times with competing risks. Stat. Med..

[30] Gu Z, Gu L, Eils R, Schlesner M, Brors B (2014). Circlize implements and enhances circular visualization in R. Bioinformatics.

[31] Gu Z, Eils R, Schlesner M (2016). Complex heatmaps reveal patterns and correlations in multidimensional genomic data. Bioinformatics.

[32] Mayakonda A, Lin DC, Assenov Y, Plass C, Koeffler HP (2018). Maftools: Efficient and comprehensive analysis of somatic variants in cancer. Genome Res..

[33] Hanzelmann S, Castelo R, Guinney J (2013). GSVA: Gene set variation analysis for microarray and RNA-seq data. BMC Bioinform..

[34] Zhang Z (2016). Reshaping and aggregating data: an introduction to reshape package. Ann. Transl. Med..

[35] Sha D (2020). Tumor mutational burden as a predictive biomarker in solid tumors. Cancer Discov..

[36] Geeleher P, Cox N, Huang RS (2014). PRRophetic: An R package for prediction of clinical chemotherapeutic response from tumor gene expression levels. PLoS ONE.

[37] Tang Q, Liang Z, Zhou Y, Huang Y (2022). Exploration of the value of combined UA, IL-6, and fPSA/tPSA in the diagnosis of prostate cancer. Comput. Math. Methods Med..

[38] Zhao R (2017). Screening of potential therapy targets for prostate cancer using integrated analysis of two gene expression profiles. Oncol. Lett..

[39] Peltanova B (2022). MRNA subtype of cancer-associated fibroblasts significantly affects key characteristics of head and neck cancer cells. Cancers (Basel).

[40] Yu L (2022). Characterization of cancer-related fibroblasts (CAF) in hepatocellular carcinoma and construction of CAF-based risk signature based on single-cell RNA-seq and bulk RNA-seq data. Front. Immunol..

[41] Wang WT, Guo CQ, Cui GH, Zhao S (2019). Correlation of plasma miR-21 and miR-93 with radiotherapy and chemotherapy efficacy and prognosis in patients with esophageal squamous cell carcinoma. World J. Gastroenterol..

[42] Ahmad I (2013). Mir143 expression inversely correlates with nuclear ERK5 immunoreactivity in clinical prostate cancer. Br. J. Cancer.

[43] Firlej V (2011). Thrombospondin-1 triggers cell migration and development of advanced prostate tumors. Cancer Res..

[44] Su W (2020). Bioinformatic analysis reveals hub genes and pathways that promote melanoma metastasis. BMC Cancer.

[45] Liu J (2018). Aberrant FGFR tyrosine kinase signaling enhances the warburg effect by reprogramming LDH isoform expression and activity in prostate cancer. Cancer Res..

[46] Huang H (2020). MIR-138-5P inhibits the progression of prostate cancer by targeting FOXC1. Mol. Genet. Genomic Med..

[47] Mohan HM (2012). Molecular pathways: The role of NR4A orphan nuclear receptors in cancer. Clin. Cancer Res..

[48] Rebello RJ, Pearson RB, Hannan RD, Furic L (2017). Therapeutic approaches targeting MYC-driven prostate cancer. Genes (Basel).

[49] Peng S (2022). UBE2S as a novel ubiquitinated regulator of P16 and beta-catenin to promote bone metastasis of prostate cancer. Int. J. Biol. Sci..

[50] Zhou M (2021). RNA-binding protein SAMD4A inhibits breast tumor angiogenesis by modulating the balance of angiogenesis program. Cancer Sci..

[51] Caspa GR, Yap LF, Paterson IC (2022). HOPX: A unique homeodomain protein in development and tumor suppression. Cancers (Basel).

[52] Tokhanbigli S (2020). Dendritic cell-based therapy using LY6E peptide with a putative role against colorectal cancer. Immunotargets Ther..

[53] Zhou M (2015). A potential signature of eight long non-coding RNAs predicts survival in patients with non-small cell lung cancer. J. Transl. Med..

[54] Costa S (2008). Importance of TP53 codon 72 and intron 3 duplication 16Bp polymorphisms in prediction of susceptibility on breast cancer. BMC Cancer.

[55] Miao J (2021). Cancer spectrum in TP53-deficient golden Syrian hamsters: A new model for Li-Fraumeni syndrome. J Carcinog..

[56] Maxwell KN (2022). Inherited TP53 variants and risk of prostate cancer. Eur. Urol..

[57] Wang G (2022). Identification of novel tumor antigens and the immune landscapes of bladder cancer patients for mRNA vaccine development. Front. Oncol..

[58] Han X, Chen J, Wang J, Xu J, Liu Y (2022). TTN mutations predict a poor prognosis in patients with thyroid cancer. Biosci. Rep..

[59] Wang Z, Wang C, Lin S, Yu X (2021). Effect of TTN mutations on immune microenvironment and efficacy of immunotherapy in lung adenocarcinoma patients. Front. Oncol..

[60] Wang W, Wang L, She J, Zhu J (2021). Examining heterogeneity of stromal cells in tumor microenvironment based on pan-cancer single-cell RNA sequencing data. Cancer Biol. Med..

[61] Tang S (2022). DHRS7 is an immune-related prognostic biomarker of KIRC and pan-cancer. Front. Genet..

[62] Jamaspishvili T (2018). Clinical implications of PTEN loss in prostate cancer. Nat. Rev. Urol..

[63] Yang C, Li Y, Yang Y, Chen Z (2020). Overview of strategies to improve therapy against tumors using natural killer cell. J. Immunol. Res..

[64] Yu Y, Wang Z, Zheng Q, Li J (2021). GREB1L overexpression correlates with prognosis and immune cell infiltration in lung adenocarcinoma. Sci. Rep..

[65] Jafari S (2020). Clinical application of immune checkpoints in targeted immunotherapy of prostate cancer. Cell. Mol. Life Sci..

