# Supplementary material for: Integrating single-cell and bulk RNA sequencing to predict prognosis and immunotherapy response in prostate cancer
Source: Sci Rep. 2023 Sep 20;13:15597. doi: 10.1038/s41598-023-42858-9 (PMC10511553; doi:10.1038/s41598-023-42858-9)
Supplement: Supplementary file 1 — Supplementary Legends. [file 41598_2023_42858_MOESM1_ESM.docx]

**Supplementary Table S1. The 463 CAFRGs.** A total of 463 CAFRGs were identified using the function "FindAllMarkers" for the high variant genes in each cluster (logFC >= 0.5, min.pct = 0.3 and diff.pct >= 0.2).

**Supplementary Table S2.** The GO analysis for different CAF clusters. The mechanisms enriched by the different CAF clusters were very different.

**Supplementary Table S3.** The KEGG analysis for different CAF clusters. The pathways enriched by the different CAF clusters were very different.

**Supplementary Table S4.** The 183 DEGs. 183 DEGs were identified between different groups with thresholds of |logFC > 1| and FDR < 0.05.

**Supplementary Table S5.** The GO analysis for 183 DEGs. BP terminology is associated with mitotic nuclear division (GO:0140014), sister chromatid segregation (GO:0000819), and mitotic sister chromatid segregation (GO:0000070); CC terminology is associated with sarcomere (GO:0030017), myofibril (GO:0030016), and contractile fiber (GO:0043292); MF terms is associated with receptor ligand activity (GO:0048018), signaling receptor activator activity (GO:0030546), CXCR chemokine receptor binding (GO:0045236).

**Supplementary Table S6.** The KEGG analysis for 183 DEGs. DEGs were highly enriched in the IL-17 signaling pathway (hsa04657), amoebiasis (hsa05146), ECM-receptor interaction (hsa04512), rheumatoid arthritis (hsa05323), staphylococcus aureus infection (hsa05150), and viral protein interaction with cytokine and cytokine receptor (hsa04061).
